# Voluntary medical male circumcision service delivery in South Africa: The economic costs and potential opportunity for private sector involvement

**DOI:** 10.1371/journal.pone.0208698

**Published:** 2018-12-17

**Authors:** Michel Tchuenche, Emmanuel Njeuhmeli, Carl Schütte, Lahla Ngubeni, Isaac Choge, Enilda Martin, Dayanund Loykissoonlal, Valerian Kiggundu, Aisha Yansaneh, Steven Forsythe

**Affiliations:** 1 Project SOAR (Supporting Operational AIDS Research), Avenir Health, Washington, DC, United States of America; 2 USAID, Washington, Washington, DC, United States of America; 3 Strategic Development Consultants, KwaZulu Natal, South Africa; 4 Avenir Health Consultant, Johannesburg, South Africa; 5 USAID, Pretoria, South Africa; 6 National Department of Health, Pretoria, South Africa; University of Oxford, UNITED KINGDOM

## Abstract

**Background:**

In 2010, the South African Government initiated a voluntary medical male circumcision (VMMC) program as a part of the country’s HIV prevention strategy based on compelling evidence that VMMC reduces men’s risk of becoming HIV infected by approximately 60%. A previous VMMC costing study at Government and PEPFAR-supported facilities noted that the lack of sufficient data from the private sector represented a gap in knowledge concerning the overall cost of scaling up VMMC services. This study, conducted in mid-2016, focused on surgical circumcision and aims to address this limitation.

**Methods:**

VMMC service delivery cost data were collected at 13 private facilities in three provinces in South Africa: Gauteng, KwaZulu-Natal, and Mpumalanga. Unit costs were calculated using a bottom-up approach by cost components, and then disaggregated by facility type and urbanization level. VMMC demand creation, and higher-level management and program support costs were not collected. The unit cost of VMMC service delivery at private facilities in South Africa was calculated as a weighted average of the unit costs at the 13 facilities.

**Key findings:**

At the average annual exchange rate of R10.83 = $1, the unit cost including training and cost of continuous quality improvement (CQI) to provide VMMC at private facilities was $137. The largest cost components were consumables (40%) and direct labor (35%). Eleven out of the 13 surveyed private sector facilities were fixed sites (with a unit cost of $142), while one was a fixed site with outreach services (with a unit cost of $156), and the last one provided services at a combination of fixed, outreach and mobile sites (with a unit cost per circumcision performed of $123). The unit cost was not substantially different based on the level of urbanization: $141, $129, and $143 at urban, peri-urban, and rural facilities, respectively.

**Conclusions:**

The private sector VMMC unit cost ($137) did not differ substantially from that at government and PEPFAR-supported facilities ($132 based on results from a similar study conducted in 2014 in South Africa at 33 sites across eight of the countries nine provinces). The two largest cost drivers, consumables and direct labor, were comparable across the two studies (75% in private facilities and 67% in public/PEPFAR-supported facilities). Results from this study provide VMMC unit cost data that had been missing and makes an important contribution to a better understanding of the costs of VMMC service delivery, enabling VMMC programs to make informed decisions regarding funding levels and scale-up strategies for VMMC in South Africa.

## Introduction

With low uptake of VMMC services and compelling evidence that VMMC is associated with men’s reduced risk of HIV acquisition through heterosexual intercourse [1,2], the South Africa National Department of Health (NDoH) introduced the procedure as an HIV prevention intervention in 2010 to complement existing HIV/AIDS prevention programs [3]. An ambitious target of circumcising 4.3 million men and boys between 2010 and 2016 was set [4]. However, by early 2015, progress toward achieving this target was lagging, with only 43% of this target being achieved [5,6].

VMMC is an integral part of the South African government's response to the HIV and AIDS epidemic [7], but there is a limited body of evidence on the unit cost of providing VMMC services. With the VMMC programs performing at less than 50% achievement of set targets [5,8], it has become necessary for the NDoH to explore other service provision modalities, such as partnering with the private sector. Private sector facilities here refer to both VMMC for-profit facilities (e.g., private clinics, hospitals and general practitioners) and not-for-profit private sector (e.g., mission clinics and hospitals, and non-governmental organizations) health facilities. The expectation is that by working synergistically with the private sector, VMMC service provision could be expanded and accelerated. In addition, it is believed that partnering with the private sector could help ease the pressure on the public sector medical infrastructure, which has been experiencing a shortage of medical doctors in recent years [9]. Indeed, Bertrand et al., noted that medical doctors in public facilities are much more likely than their counterparts in other services to burn out and leave the VMMC program [9]. Thus, harnessing the human resources in the private sector could contribute to mitigating the impact of VMMC staff turnover in the government-supported facilities. Private sector facilities therefore represent a strategic potential to increase access to VMMC services as well as attract clients who may not necessarily go to public health facilities for services.

Against the backdrop of the slow progress in VMMC coverage, the NDoH requested a rapid yet robust study to estimate the unit cost of providing VMMC in South Africa. The study to determine the cost of providing a comprehensive set of services for VMMC in South Africa at facilities supported both by the government and the President's Emergency Plan for AIDS Relief (PEPFAR) was completed in 2015 [10]. The study noted that the lack of sufficient data from the private sector represented a gap in knowledge concerning the overall cost of scaling up VMMC services in South Africa. As a result, the NDoH and PEPFAR provided support for the present study to undertake a detailed economic cost analysis to contribute to a better estimate of the resources required to further scale up the provision of VMMC services in South Africa by engaging with the private sector.

The objectives of this study were therefore to: 1) determine the private facility unit cost of providing medical circumcision by mode of service delivery (fixed, mobile and outreach sites), geography (provinces), and by level of urbanization (urban, peri-urban and rural); and *2)* assess cost drivers and cost variances across the provinces and different VMMC service delivery modes.

## Methods

### Ethical considerations

Ethical approvals for the study were obtained from the Human Research Ethics Committee (Medical) of the University of the Witwatersrand, South Africa, and the Health Media Lab’s Institutional Review Board in Washington DC, registered with Federalwide Assurance approval by the U.S. Department of Health and Human Services, Office of Human Research Protections.

Due to confidentiality, the names of the facilities are coded (Table 1). Verbal informed consent was obtained from key informants at the facility, which largely included VMMC program managers and financial managers.

**Table 1:** VMMC facilities surveyed.

Province	Facility code	Service delivery model	Location	Total # of VMMC clients (12 months)
Gauteng	G1	Fixed	Urban	4,949
	G2	Fixed	Urban	2,678
	G3	Fixed	Peri-urban	1,758
	G4	Fixed	Peri-urban	1,235
	G5	Fixed	Peri-urban	8,378
	G6	Fixed, outreach & mobile	Urban	1,080
KwaZulu-Natal	K1	Fixed	Peri-urban	216
	K2	Fixed	Urban	1,570
	K3	Fixed with outreach	Urban	575
	K4	Fixed	Urban	1,300
Mpumalanga	M1	Fixed	Rural	3,765
	M2	Fixed	Rural	2,770
	M3	Fixed	Urban	1,217

### Data collection and key assumptions

Using an ingredients-based approach where all relevant inputs are listed [11,12], a data collection instrument was developed. The study team conducted on average two site visits for data collection through interviews of facility staff members involved in the provision of VMMC services. Where data were incomplete, the research team requested clarifications. Selection of study sites was conducted in consultation with NDoH, PEPFAR and other key stakeholders. Three provinces—Gauteng, KwaZulu-Natal and Mpumalanga—were selected with the aim of conducting data collection in at least three facilities in each province. Primary selection criteria were driven by limited project budget and stronger stakeholder engagement in these three provinces. A process was undertaken to generate a list of VMMC private service facilities who routinely provided VMMC services for at least six months prior to the study. Most facilities approached (20 in total) were willing to participate in this study, though some were concerned about the time needed to complete the survey that may affect attending to their general clientele. Ultimately, 13 facilities (Table 1) agreed to provide data. Two of the facilities were not-for-profit (one supported by an NGO and the other one supported by a mission) while the remaining 11 were for-profit sites.

Financial expenditure and human resource data required for estimating unit cost per VMMC beneficiary in the private sector were collected retrospectively between June and November 2016 from 13 facilities across the three selected provinces. Data were collected for the most recent 12-month period in 2015/2016 for which program data were available (for most facilities this was June 2015 to May 2016). Key VMMC personnel interviews were semi-structured and conducted among facility VMMC program managers, finance officers, general practitioners and/or medical officers conducting the surgical procedure.

### Human resources

At each facility, information was collected on direct labor (clinical personnel such as general practitioners, clinical associates, and nurses) and indirect labor (staff who do not provide any direct VMMC services to clients). Information on the employment status (permanent versus contracted staff) was collected for both direct and indirect staff members. Additional information collected included the number of personnel, their salaries, and the level of effort (proportion of time) allocated to VMMC. When indirect staff members’ time was not provided, then the proportion of VMMC clients relative to the total client volume at the facility was used to approximate indirect staff time allocated to the VMMC program.

### Medications and other consumables

Information was collected on medicines and other consumables, the quantity distributed to each VMMC client and the cost of each item. Priority was given to sites that provided their own estimates of input costs. However, in cases where costs of medications and/or consumables were not available, the study team used the information collected from a variety of sources in 2015, including the Supply Chain Management System, Clinton Health Access Initiative (CHAI), and PEPFAR as well as from Northdale Hospital and the Voluntary Medical Male Circumcision Centre of Excellence in KwaZulu-Natal [10]. PEPFAR-provided costs of $17.80 per male circumcision kit (i.e., bundle set of devices used to perform a male circumcision and post procedure care) was attributed to all facilities because those that indicated they did not purchase the male circumcision kits from PEPFAR provided no alternative cost.

### Equipment and furniture

Facilities were asked to provide a list of all equipment and furniture utilized in the screening/review room, the counseling area, the operating theatre, and/or any relevant sterilization areas. In addition, facilities were asked to identify general equipment that was used as part of the VMMC program. Each facility then provided information on the number of items utilized and the estimated proportion of time that equipment and/or furniture was utilized for VMMC. The useful life of equipment and furniture was obtained from WHO-CHOICE (Choosing Interventions that are Cost-Effective) [13]. When useful life estimates were not available from WHO-CHOICE, data was collected from a recent circumcision costing study at government and PEPFAR supported facilities in South Africa [10].

### Vehicles

Vehicles were used for transportation of supplies and staff to VMMC outreach sites and very rarely for the transportation of clients. For facilities offering other services besides male circumcision, only vehicles used by the facility as part of the VMMC program were recorded as well as the proportion of time the vehicles were used by the VMMC program. Vehicle costs were amortized, and the proportion allocated to the VMMC program was determined based on the number of VMMC clients, relative to the total number of clients at the facility.

Annual overhead costs included a range of items: costs associated with utilities (water, electricity, internet, telephone, waste management, cleaning services, etc.) and the rental cost of the facility. Overhead costs were apportioned to the VMMC program based on the number of VMMC clients, relative to the total number of clients seen at the facility.

### Continuous quality improvement (CQI)

Monitoring the quality of VMMC service provision is key to the success and scale-up of VMMC program Given that the NDoH seeks to involve the private sector in reaching its ambitious VMMC targets, it is anticipated that CQI will be extended to VMMC private facilities. For this reason, an average cost of $17.64 to provide CQI for PEPFAR partners in South Africa was added to the calculated VMMC unit cost, based on comparable services offered at public and PEPFAR-supported facilities [10].

### Training

VMMC personnel training costs collected was insufficient to estimate the per circumcision training cost because the number of personnel trained or retrained was missing for most facilities. For this reason, the average national-level data on the cost of training per circumcision of $2.31 reported in the previous study [10] was used. It is important to note that private providers undergo the same training (provided by the Centre for HIV and AIDS Prevention Studies (CHAPS), VMMC Centre of Excellence (KZN DOH) or Jhpiego as personnel in public facilities. Thus, using the cost reported in [10] is viable.

### Data analysis

To estimate the private sector unit cost of providing VMMC services, key assumptions were made.

- Following the completion of the data cleaning process, the study team entered data from the 13 facility surveys into the developed costing model: an Excel spreadsheet customized to calculate the unit cost of VMMC in South Africa as a key output [14].
- The proportion of VMMC clients relative to the total client volume at the facility was used to apportion indirect staff time and other overheads to the cost of VMMC service provision. Using the number of VMMC clients as weight, the unit cost was then calculated as weighted average of the 13 facilities.

**Fig 1:**
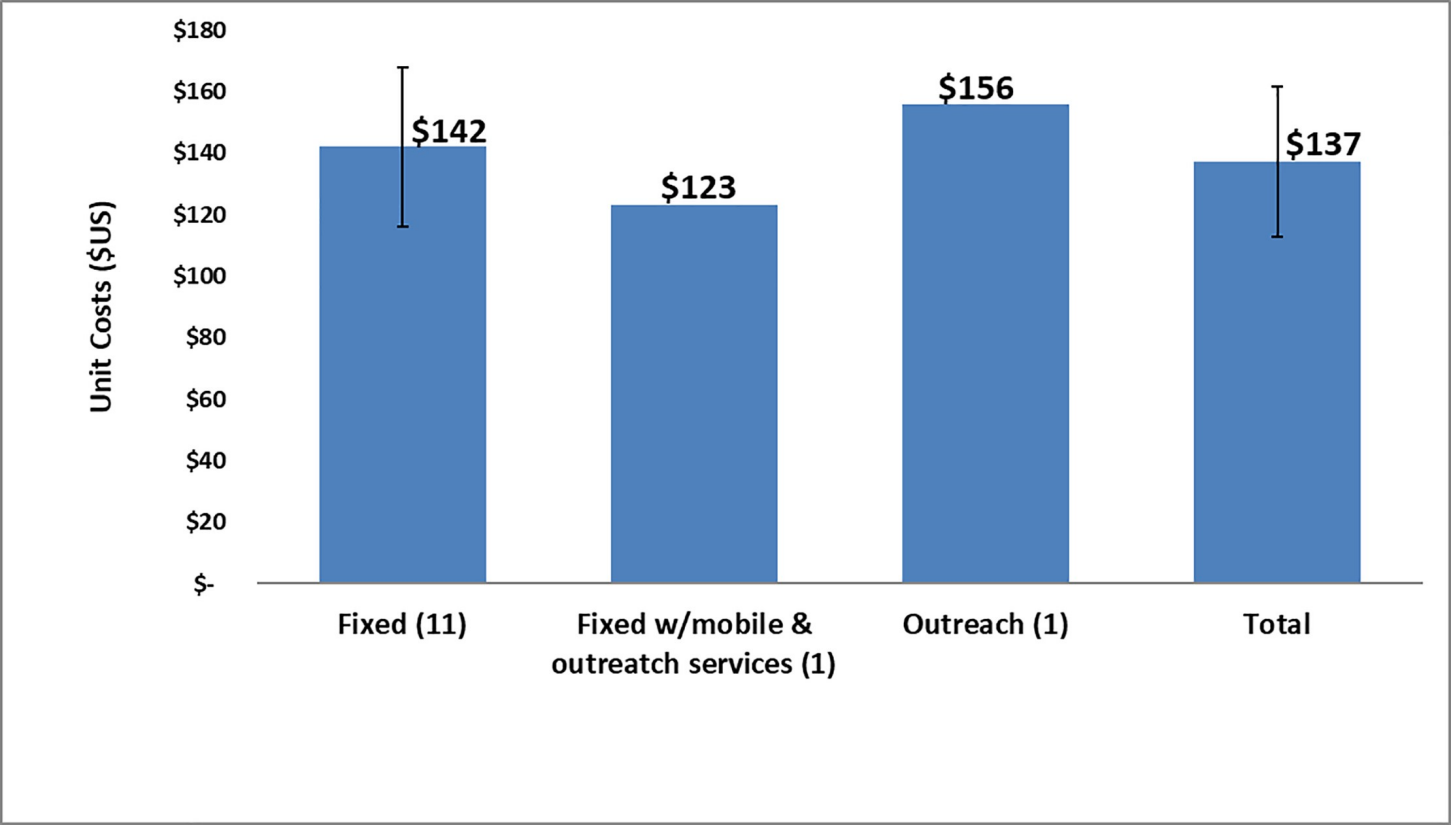
Unit costs by mode of service delivery.

**Fig 2:**
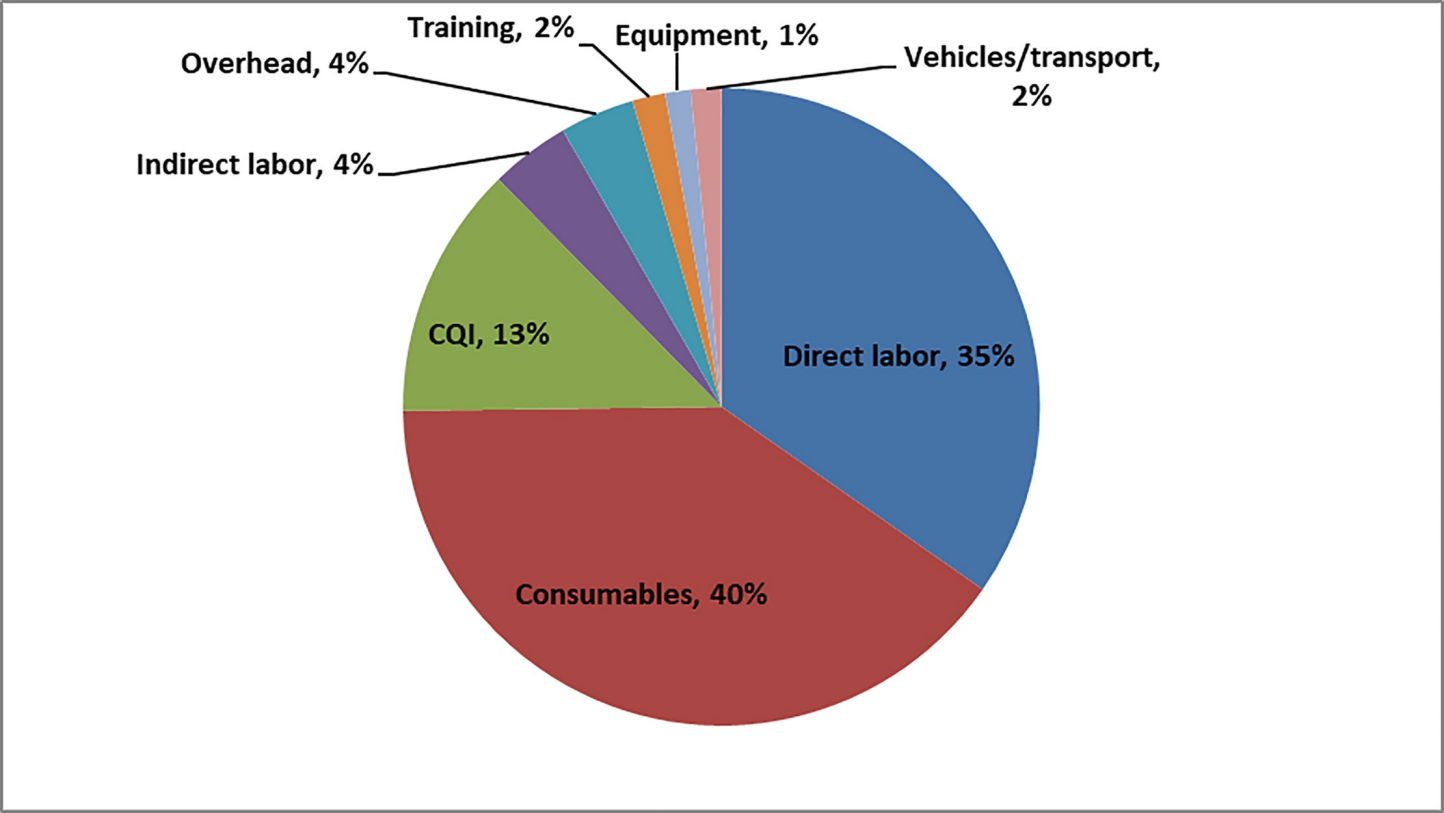
VMMC unit cost by major cost category.

**Fig 3:**
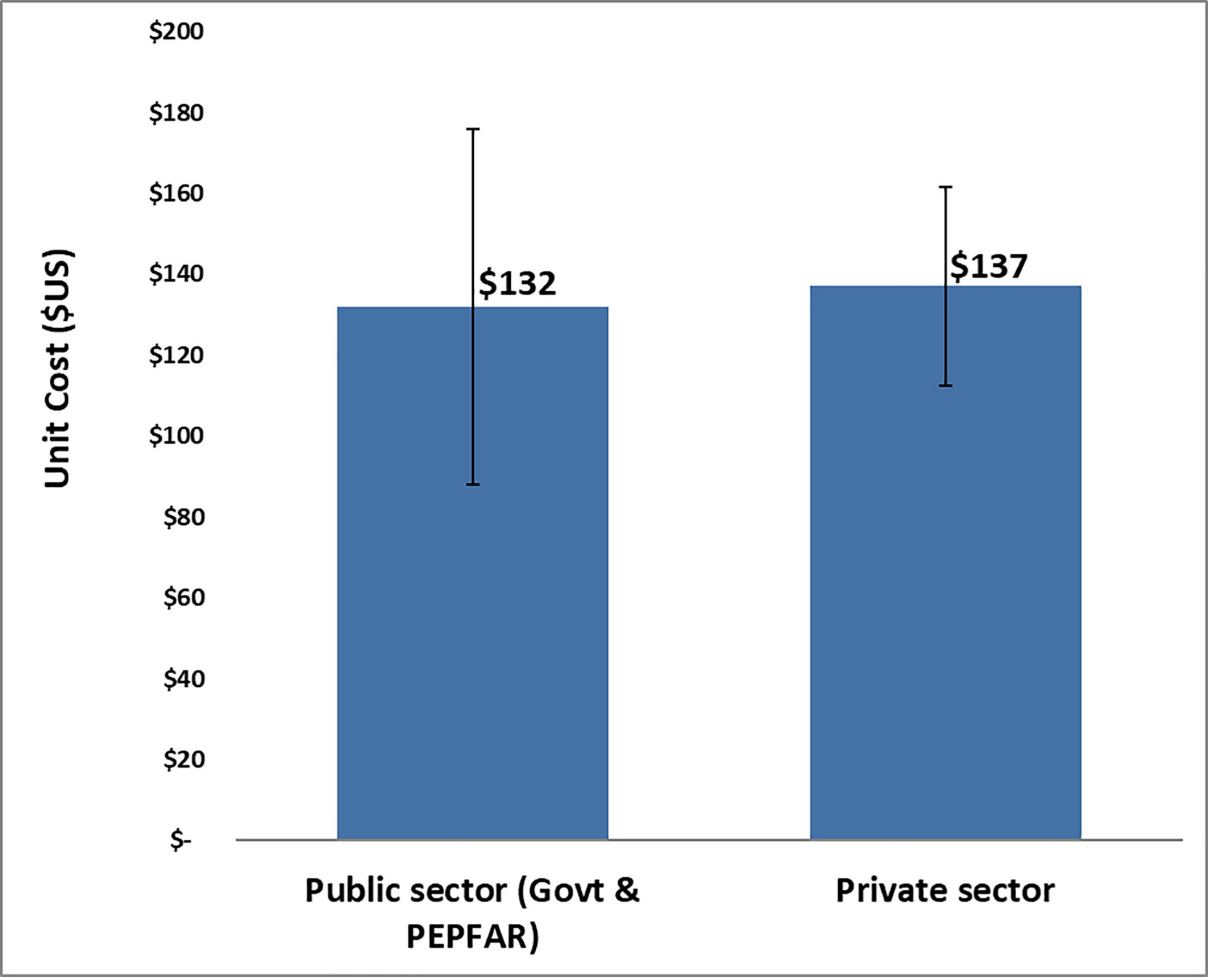
Unit cost by province.

**Fig 4:**
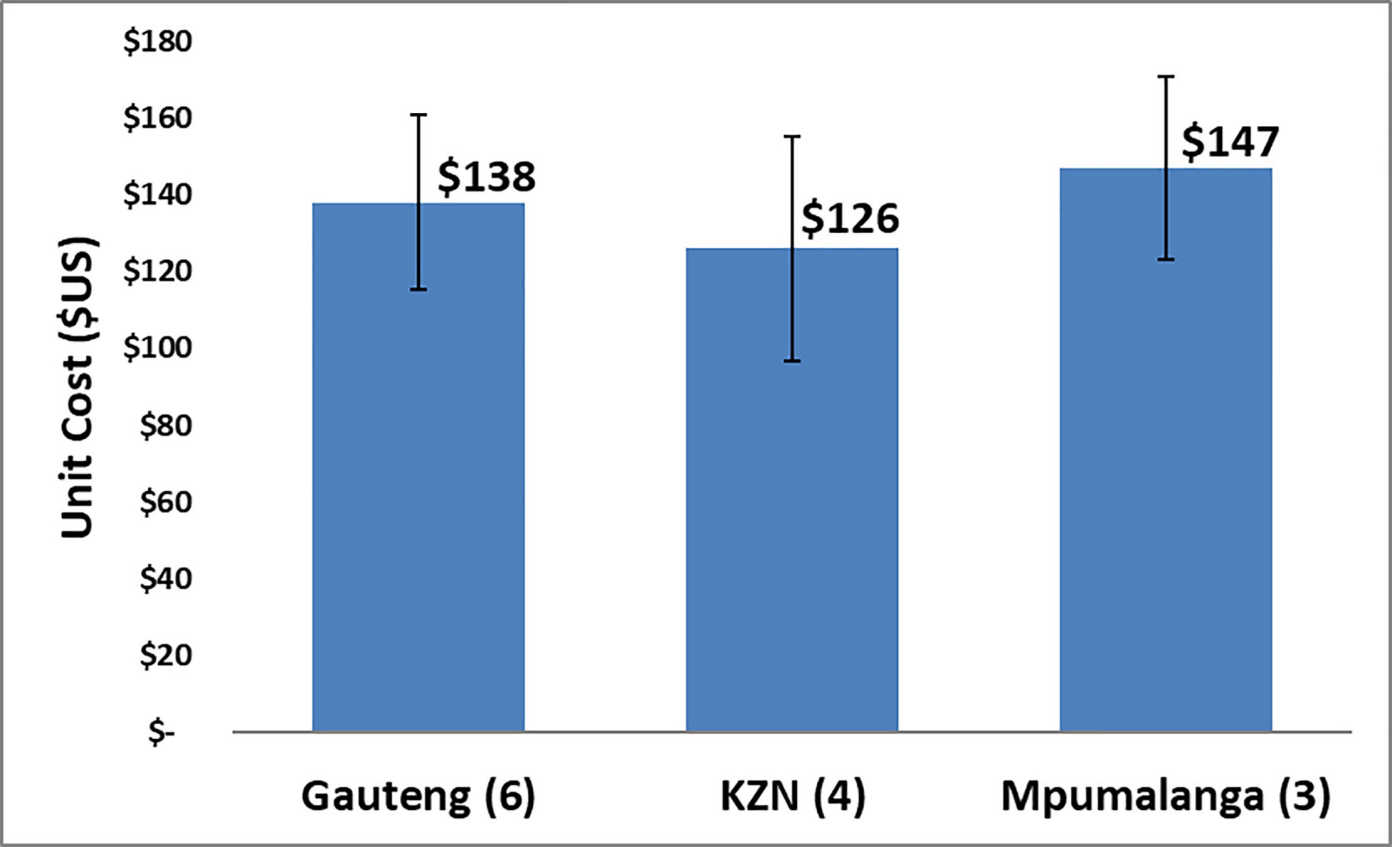
Unit cost by province urbanization level.

**Fig 5:**
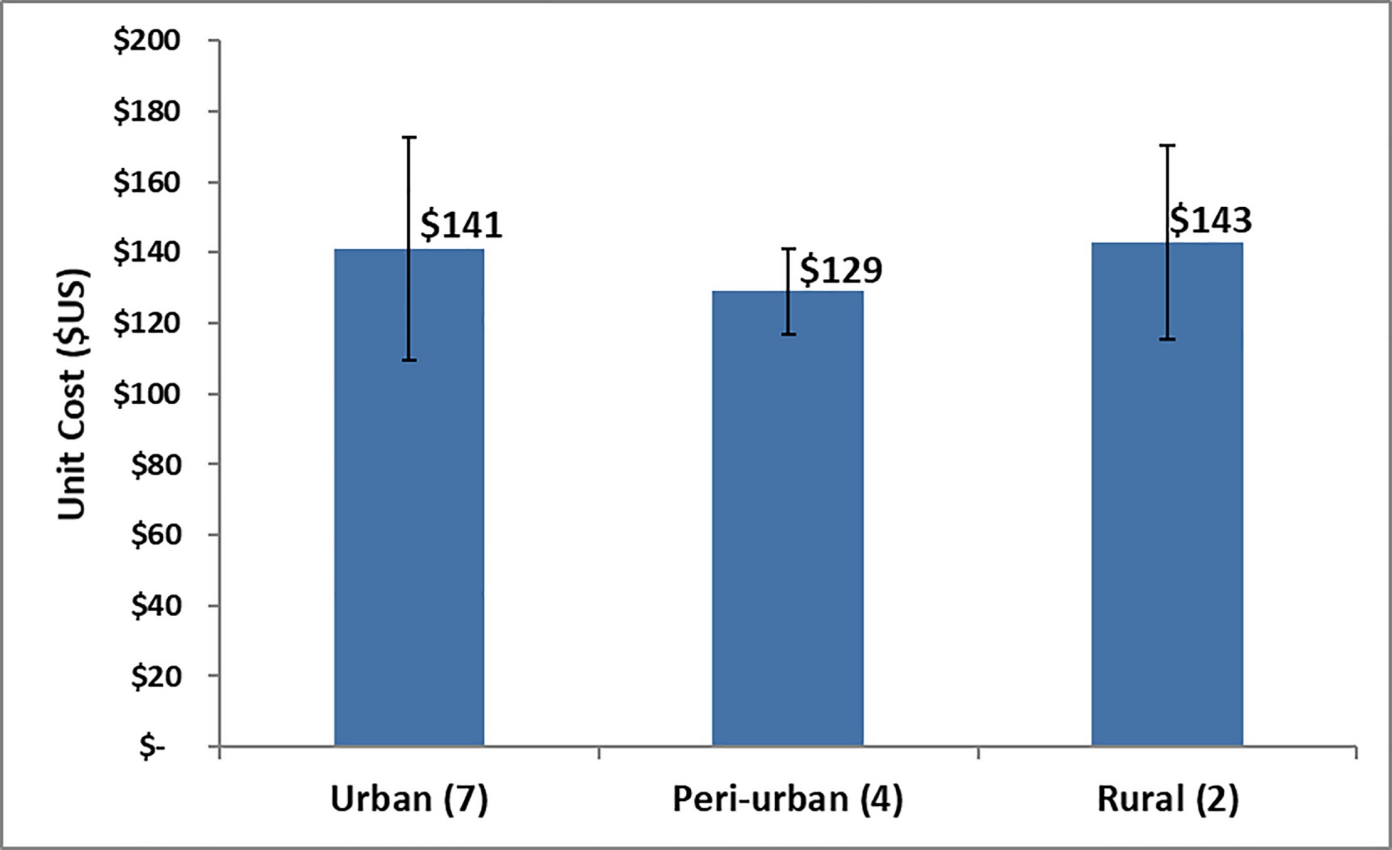
Unit cost by facility scale of operations.

**Fig 6:**
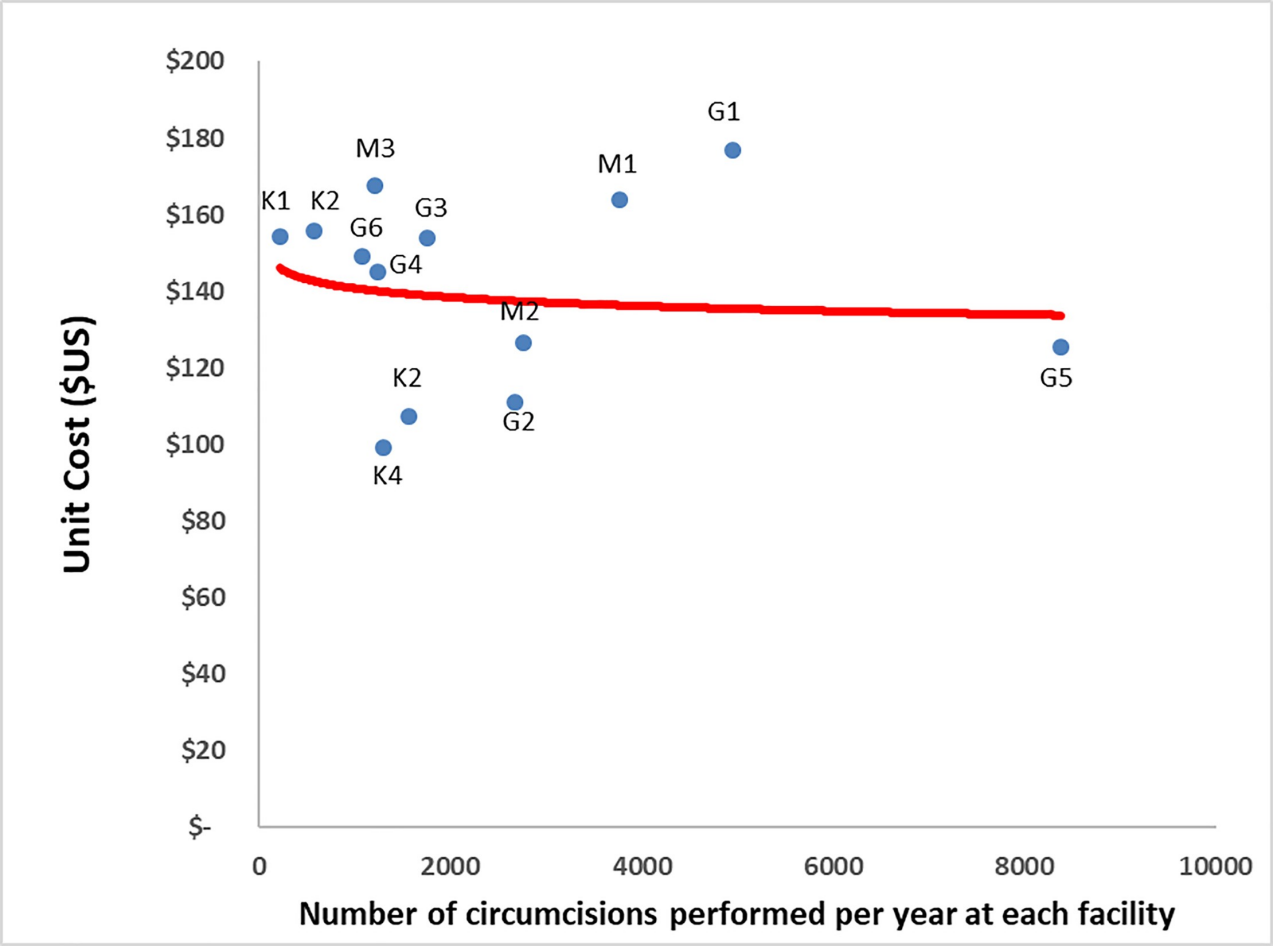
Unit cost variation across facilities.

**Fig 7:**
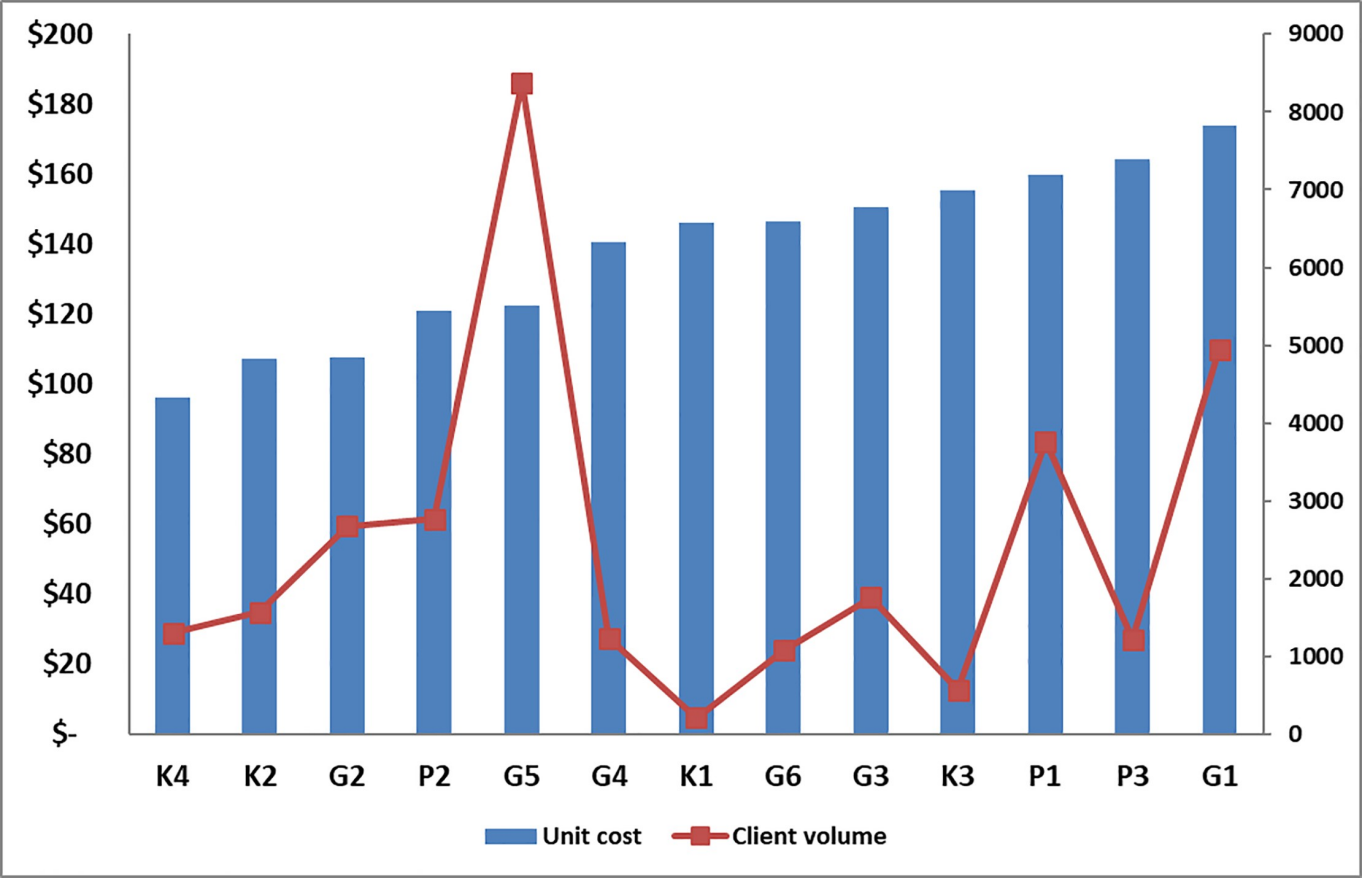
Public and private sectors unit cost comparison.

Results are graphically represented in Figs 1–7. The error bars in Figs 1, 3, 4 and 5 represent the standard deviation (SD) values used in both directions.

## Results

From the cost data collected, the weighted average cost per circumcision performed at the 13 private facilities was estimated to be $137.29 (for convenience, the value $137 is used throughout the text). This unit cost included the average training cost ($2.31) as well as CQI cost per circumcision performed ($17.64) collected at government and PEPFAR-supported sites [10]. Table 2 below provides the unit cost by cost category.

**Table 2:** VMMC unit costs.

	Cost (US$)	%
**Direct cost**		
Direct labor	$ 47.64	35%
Consumables	$ 55.06	40%
*Sub-total*	*$ 102*.*70*	*75%*
**Indirect cost**		
Indirect labor	$ 5.55	4%
Overhead	$ 5.18	4%
Equipment	$ 1.78	1%
Vehicles/transport	$ 2.14	2%
CQI	$ 17.64	13%
Training	$ 2.31	2%
*Sub-total*	*$ 34*.*59*	*25%*
**Total**	$ 137.29	100%

### Unit cost by mode of service delivery

Fig 1 shows the unit cost difference between the types of facility. With only one fixed site with outreach services and one fixed site with both mobile and outreach services that participated in the study, it is not possible to draw any meaningful conclusion on the difference of unit cost by mode of service delivery in the private sector.

### Unit cost by cost category

Fig 2 depicts the major VMMC cost categories in the private sector.

### Unit cost by province

Fig 3 provides a breakdown of unit costs by province. The number of VMMC sites within a given province is indicated in brackets next to the province’s name.

### Cost by urbanization level (urban, peri-urban, and rural)

Each site was classified depending on the level of urbanization in the communities where the facility operates: urban, peri-urban, or rural areas in the province, with the unit costs of performing VMMC respectively $141, $129, and $143 (Fig 4).

### Unit cost by scale

There are sites with higher numbers of VMMC clients and lower unit cost (e.g., Sites G2, G5 and M2), while some lower-volume sites also have a low unit cost (e.g., Sites K2 and K4). On the other hand, some higher-volume sites have a high unit cost (e.g., Sites G1 and M1). Facilities are arranged according to their VMMC client volume, from lowest to the highest annual number of circumcisions performed per year at each facility. The red line in Fig 5 generated using Excel statistical functions represents the logarithmic trendline which depicts the relationship between the VMMC unit cost and the number of circumcisions performed per year at each facility (or the scale of the operation). There is a slightly noticeable small inverse relationship between annual client volume and unit cost despite several outliers. Consequently, scale of operation does not fully explain the variation in unit cost given the variations around the mean unit cost.

In Fig 6, unit costs are sequentially arranged from lowest $96 to highest $174.

### Unit cost comparison—Public vs private facilities

The average unit cost per circumcision performed at the 13 private facilities in only three out of the nine provinces (mean $137, SD $25) was a little higher than in the 33 public facilities (in 8 provinces) previously studied (mean $132, SD $44)–difference $5 (95% confidence interval -$12 to $32). It is important to emphasize again that the narrow geographical focus was due to limited project budget and stronger stakeholder engagement in these three purposively selected provinces. Fig 7 depicts this cost comparison with the up and down error bars representing the standard deviations. However, the average client volume per facility was higher at private facilities–2,422 clients on average at the 13 private facilities surveyed compared to the 1,978 clients on average at the 33 public and PEPFAR-supported sites surveyed in [10].

Unit cost components are comparable across the public and private sectors. Consumables and direct labor in private facilities accounted respectively for 40% and 35% versus 24% and 43% in public facilities. Unit cost did not differ in both the private and public sectors in KwaZulu-Natal, respectively $126 and $115, while in Gauteng this was $138 vs $123 and Mpumalanga $147 vs $164.

### Study limitations

This study is not exhaustive, and some limitations are noted below. Data were collected at 13 facilities from only three of South Africa’s nine provinces. Stakeholder engagement was paramount for private sector buy-in and to gain their consent for study participation. Of the 20 sites that were approached with request to participate in this analysis, only 13 successfully provided the required data. Reasons given for non-participation was either the facility had been operating for less than six months, data quality issues had not been resolved, or the data collection fell within a busy period at the facility. Strong VMMC stakeholder engagement influenced the province selection and sites were not randomly selected due to resource limitations. A larger sample size and more variation by facility type and service delivery model could have provided better opportunities to compare unit costs across all the nine provinces. Such an exhaustive study was however not feasible, however, given time and resource limitations. The results therefore may not be fully representative of all private sector VMMC facilities throughout South Africa.

Study researchers did not do a time-motion analysis of how staff spent their time on the VMMC program at private facilities. Instead, respondents (i.e., the key VMMC personnel interviews such as VMMC program managers, finance officers, general practitioners and/or medical officers conducting the surgical procedure) were asked to provide a general level of effort. This approach could potentially lead to a recall bias, the extent of which is unknown because assessing this was beyond the scope of this study. Likewise, while every effort was made to obtain all information about resources used at facilities, the interview process required respondents to accurately recall and identify direct and indirect resources.

The process of mobilizing and motivating men to access VMMC services is known as demand creation [10]. This is key to the successful implementation and scale-up of a VMMC program. In some cases, private sector facilities readily engaged in demand creation through newspaper adverts and other demand creation approaches. However, having private sector facilities continuously invest in demand creation is only likely to occur if they observe that the demand creation efforts are successfully increasing their client volume, and/or if they have a financial incentive to expand their clientele. It was noted, for example, that some private facilities engage in VMMC provision so that they can also offer clients other services and ideally assure that the client makes return visits for other health issues. Since our study questionnaire was not tailored to link spending on demand creation to the increased number of clients, demand creation expenditure was excluded from the unit cost calculation. George et al., [7] in a recent study to estimate the cost of demand creation activities and VMMC targeting school-going adolescents in KwaZulu-Natal, South Africa, found that demand creation activities accounted for 32% of the total VMMC unit cost.

## Discussion

This study provides evidence on VMMC costs by private facilities in South Africa and adds to a more comprehensive understanding of VMMC service costs across public (Government/PEFAR) and private sectors. The unit cost of circumcisions performed at the 13 selected private facilities was estimated to be $137, compared to the cost of providing VMMC services in South Africa at government and PEPFAR-supported facilities estimated at $132 per circumcision performed [10].

One site (G5) had the highest client volume 8,378. If this site is excluded from the analysis, the per circumcision unit cost slightly increases to $143. Mpumalanga, with two rural sites and one urban site in the sample, has the highest unit cost, $147. These two sites have more contracted staff (whose labor costs are higher than public or private permanent staff, than other surveyed private facilities in Gauteng and KwaZulu-Natal). This same trend which seems to indicate that contracted personnel are potentially more expensive was also observed in the costing study at government and PEPFAR supported facilities [10].

Direct costs, in the form of consumables and direct labor were the first and second most important cost drivers of VMMC unit cost in the private sector. Direct costs were comparable across private and public (non-profit) facilities, accounting respectively for 75% and 67% of total cost [10]. Our results are also in agreement with a regional analysis which found that the two main cost drivers associated with providing VMMC in sub-Saharan Africa are personnel and consumables [15]. The difference in the cost of direct labor (35% in private facilities vs. 43% in public/non-profit facilities) could be attributable to the higher volume of clients at private facilities, creating economies of scale. However, there is no clear relationship between volume and unit cost (Fig 6) as the small sample size with large variation around the weighted average unit cost may be masking the existence of such a relationship.

Although unit cost components are comparable across the public and private sector, there is a difference in per patient overhead cost of the outreach facility compared to the overhead cost from the other two modes of service delivery because this outreach facility operates VMMC services once a week with a low annual client volume. It is important to note that costs for outreach and mobile services in the private sector should be viewed with caution as costs were obtained from only two sites (one each– 1 site with outreach and the other one with mobile services).

The average annual number of circumcisions performed at private facilities is 2,422 clients per year versus 1,978 at government and PEPFAR-support facilities. Thus, private facilities appear to be performing about 24% more circumcisions than the public sector and PEPFAR-supported non-profit facilities, at approximately the same cost per circumcision performed (only a 4% increase in overall cost). There was wide variation of unit cost and patient volume across all the 13 facilities surveyed (Fig 7).

Private facilities noted that demand creation is critical to bringing more clients into their practice. However, a challenge they face is financing demand creation activities. About one-third of the facilities surveyed indicated that they spent some funds on demand creation, but they recruit community mobilizers only on a contract basis as the need arises. Private facilities potentially benefit from population-wide demand creation activities undertaken by the public sector, but the extent of the benefit is not known and is not within the scope of this study but would be an area needing further investigation. If the public sector can work synergistically with the private sector to better assess their demand creation needs, it should be feasible to further increase demand for private sector VMMC services without significantly increasing the cost of service delivery as well as the overall cost of VMMC demand creation. However, for the reasons indicated above (service provision not adequately linked to demand creation activities), demand creation expenditures were not included in the unit cost calculation for either the public/PEPFAR or the private sector.

About 50% of private facilities commented on their reimbursement cost for VMMC services, noting a low compensation level received per circumcision performed (out of which they provide transport incentives to their clients). Though not directly related to the data collected and analyzed, this is an important issue for the private facilities and a potential barrier for scaling up VMMC services in the private sector. It is the costing study team’s hope that this study could be a catalyst for a public-private partnership engagement with private insurance companies and private facilities in South Africa which could develop a standardized VMMC tariff structure and provide a platform for discussions on the reimbursement of private sector facilities for circumcisions of uninsured clients.

It has been reported that VMMC clients accessing VMMC services at government and PEPFAR supported facilities do incur up to US$9.20 indirect out-of-pocket costs for transportation or foregone income [16]. Investigation of clients’ out-of-pocket expenditure for transportation or foregone income travelling to private facilities was beyond the scope of this study.

In a nutshell, results from this study could provide potential guidance to VMMC stakeholders (e.g., health policymakers and VMMC program managers) at the national, provincial, and district level—to make informed VMMC strategic planning decisions regarding the funding and scale-up of VMMC in South Africa in partnership with the private sector. In fact, the government of South Africa should consider utilizing the services of private VMMC facilities to meet its ambitious target and to sustain the VMMC program in the long run.
